# Activin A Stimulates Aromatase via the ALK4-Smad Pathway in Endometriosis

**DOI:** 10.1155/2016/5791510

**Published:** 2016-10-19

**Authors:** Juan Zheng, Juan Qu, Pinhong Lu, Zhen Hou, Yugui Cui, Yundong Mao, Xiaochen Qi, Hui Ji, Jiayin Liu

**Affiliations:** ^1^State Key Laboratory of Reproductive Medicine, Center of Clinical Reproductive Medicine, First Affiliated Hospital, Nanjing Medical University, Nanjing 210029, China; ^2^Center of Reproductive Medicine, 174th Hospital of PLA, Xiamen 361000, China

## Abstract

Endometriosis is an estrogen-dependent disease. We previously found that the expression of Activin A was upregulated in the peritoneal fluid of patients with endometriosis. The results of the present study indicated that Activin A induced estradiol secretion and P450arom expression in endometrial stromal cells (ESCs) derived from endometriosis patients. The mechanism of estrogenic synthesis was regulated by the Activin-Smad pathway in endometrial lesions. The data showed that the effect of Activin A on ESCs was partially abrogated by pretreatment with an inhibitor of ALK4 (the type I receptor, ActRIB) and Smad4-siRNA. Cumulatively, these data suggest that Activin A promotes the secretion of estradiol from ESCs by increasing the expression of P450arom via the ALK4-Smad pathway. These findings indicate the ALK4-Smad pathway may promote ectopic lesion survival and development.

## 1. Introduction

Endometriosis is one of the most common gynecological disorders and affects 10% of reproductive-aged women. The disease is defined by the presence of functional endometrial tissue outside the uterine cavity. The tissue is primarily located on the pelvic peritoneum and ovaries. The main clinical features include chronic pelvic pain, dyspareunia, and infertility [1]. Endometriosis has been traditionally defined as an estrogen-responsive disease [2–5]. In addition, endometrial lesions display increased estradiol biosynthesis and decreased estradiol inactivation compared to normal endometria [6, 7].

Estrogen in the circulation of women suffering endometriosis arises from three sources. First, estradiol secreted by the ovary reaches endometrial tissues through circulation. The follicular rupture during each ovulation causes the release of large amounts of estradiol directly onto pelvic implants [8]. Second, the aromatase P450 that resides in adipose tissues and skin catalyzes the conversion of circulating androstenedione to estrone [8]. This circulating androstenedione travels to endometrial tissues and is converted locally to estradiol. Third, endometrial stromal cells may synthesize E_2_ *de novo* from cholesterol by the classical steroidogenic pathway because they express all components required for the steroidogenic cascade, which includes P450arom for estrogen biosynthesis. Peripheral or local P450arom activity may be particularly important in the persistence of endometriosis [8]. A molecular link between inflammation and estrogen production in endometrial tissues has been previously reported [7, 9, 10]. The estradiol production is mediated by a positive feedback cycle that favors the overexpression of key steroidogenic genes, including P450arom, which increases the local production of estradiol and promotes the secretion of transforming growth factor-beta (TGF-*β*) and PGE_2_ in endometrial tissues [7].

Our previous studies assessed the expression of different cytokines using cytokine arrays of peritoneal fluids taken from subjects with minimal/mild endometriosis, moderate/severe endometriosis, and controls [11]. Activin A was one of the upregulated cytokines in the women with endometriosis [11]. It is a highly conserved member of the TGF-*β* superfamily. This ligand initiates signal transduction pathways that are critical for reproductive functions and development [12]. Activin A binds to the heteromeric receptor complex consisting of a type I (ActRIA and ActRIB, ALK4) and type II (ActRIIA and ActRIIB) receptors [12]. The type I receptors are activated by phosphorylation and subsequently initiate the Smad signaling pathway. The phosphorylated receptor Smads (R-Smads) bind to the common Smad (co-Smad, Smad4) and enter the nucleus as a complex. In the nucleus, the Smad signaling complex regulates the transcription of target genes through interactions with transcriptional regulatory elements [12]. Conversely, the inhibitory Smads (Smad7) have been shown to prevent the activation of the Smad signaling pathway by occupying ligand-activated type I receptor (ActRIB, ALK4) and interfering with the phosphorylation of R-Smad. The inhibitor Smads interfere with the activation of TGF-*β* signaling [13–15].

Several studies have shown that Activin signaling through Activin receptor-like kinase 4 (ALK4) stimulates P450arom activity in the ovarian granulosa cells and placental trophoblast cells [16, 17]. Furthermore, Activin stimulates* CYP19A* gene expression in human ovarian granulosa cell-like KGN cells via the Smad2 signaling pathway. Activin signaling through the ActRIB-Smad2 pathway plays a pivotal role in* CYP19A* expression and follicular development [18]. However, little was known about the underlying mechanism of Activin signaling in the pathogenesis of endometriosis. We hypothesized that Activin A stimulates endometrial stromal cells in endometriosis by inducing the expression of P450arom via the ALK4-Smads signaling pathway. The present study was designed to investigate whether the Activin-Smads signaling pathway is functionally present and upregulated in the endometrial lesions. This study also examined whether the activated Activin-Smads signaling pathway can promote the synthesis of estrogen in endometrial stromal cells. Furthering our understanding of the signaling mechanisms regulating estrogen biosynthesis in endometriotic lesions may lead to the discovery of new diagnostic and therapeutic targets for endometriosis.

## 2. Materials and Methods

### 2.1. Patient Selection and Characterization

Sixty-five women scheduled for laparoscopy for infertility were recruited to participate in this study after providing written informed consent. The study was approved by the Medical Research Review Board of First Affiliated Hospital, Nanjing Medical University (Nanjing, China). All of the patients had no active pelvic inflammatory disease, hydrosalpinges, or any autoimmune disease. They had no history of endometrial hyperplasia or neoplasia. The subjects had not received any anti-inflammatory or hormonal medications or received a hysterosalpingography in the past three months. All endometrial biopsies were in the proliferative phase which is confirmed with a pathological examination of the endometrial biopsy by the method described previously [19].

The diagnosis of endometriosis was confirmed in 55 women (mean ± SD of 30.1 ± 3.21 years of age) by the presence of glands and stromas in the endometrial lesions. Endometriosis was diagnosed and staged according to the revised American Society for Reproductive Medicine (rASRM) classification by visual inspection of the pelvis [20]. Thirty women had the minimal/mild disease (AFS stages I-II, 29.9 ± 3.4 years old) with red endometriotic lesions, such as red vesicles, red flame-like lesions, or gland-like lesions. Twenty-five women had the moderate/severe disease (AFS stages III-IV, 30.5 ± 2.96 years old). Ten women (31.9 ± 1.9 years old) that received a laparoscopic examination for infertility without endometriosis served as the control group. There were no endometrial lesions, active pelvic inflammatory diseases, or hydrosalpinges observed via laparoscopy in the control group.

### 2.2. Preparation of Endometrial Stromal Cells

The endometrial tissues were obtained via dilation and curettage during surgery. Immediately after collection, small portions of endometrial tissues were prepared for endometrial stromal cells. Endometrial stromal cells (ESCs) were separated from the epithelial glands by digesting the tissue fragments with collagenase as previously described [21]. The isolated ESCs were cultured in Dulbecco modified Eagle medium F-12 (DMEM/F12; Sigma-Aldrich, St. Louis, Missouri) medium containing 10% fetal bovine serum (FBS; Gibco, Australia) until reaching confluence. After 24 h of culturing, the cells were characterized using immunofluorescence microscopy with antibodies to cytokeratin 19 and vimentin as previously described [22]. Vimentin staining of the sample preparation demonstrated that the purity of the ESCs is more than 95%. ESCs were seeded in six-well culture plates before use in experiments.

### 2.3. Experimental Design

ESCs from patients with endometriosis were cultured in DMEM/F12 until 80–90% confluence. Then, ESCs were treated with various concentrations (0, 2.5, 25, and 50 ng/mL) of Activin A for different time (0 h, 2 h, 12 h, and 24 h). Activin A was purchased from R&D Systems (Minneapolis, MN, USA). Real-Time PCR and Western blot were used to determine the expression of P450arom. ESCs from normal endometrium and patients with endometriosis were stimulated with Activin A (25 ng/mL) or testosterone (100 ng/mL) for 24 h. After treatment, the supernatants of the cell culture were collected and frozen in −80°C for the detection of concentrations of estradiol.

ESCs from patients with endometriosis were treated with 25 ng/mL Activin A for 5 min, 15 min, and 1 h. Western blot and immunofluorescence were used to determine the expression of p-Smad3 and Smad3.

After pretreatment with an inhibitor of ALK4 (the type I receptor, ActRIB) and Smad4-siRNA, real-time PCR and Western blot were used to determine the change of P450arom expression in ESCs from patients with endometriosis stimulated with 25 ng/mL Activin A. Estradiol secretion was also detected.

Each study was repeated with three independent samples, and each experiment was performed in triplicate.

### 2.4. Preparation of Cell Fraction

The separation and preparation of cytoplasmic and nuclear extracts from ESCs were performed using a commercial kit following the manufacturer's instructions (KeyGEN Biotech, Nanjing, China). In brief, cytoplasmic extraction reagents (A and B) were added to a cell pellet to disrupt cell membranes to release cytoplasmic contents. The integrity of the nuclei extraction was verified by the examination with a light microscope. The samples were lysed with a nuclear extraction reagent to yield the nuclear fraction by centrifugation. Immunoblot analysis was used to assess the adequacy of nuclear purification by measuring LaminA/C (nuclear protein) and* GAPDH* (cytoplasmic protein) content. The protein concentrations were determined using the bicinchoninic acid (BCA) protein assay kit (KeyGEN Biotech).

### 2.5. Real-Time PCR

Total cellular RNA was isolated from cells using Trizol (Invitrogen Corporation, Carlsbad, CA, USA). One microgram of total RNA was subjected to reverse transcription using PrimeScript RT-PCR Kit (Takara Shuzo Co. Ltd., Kyoto, Japan). For the PCR reaction, cDNAs were used as templates to coamplify aromatase and* GAPDH* mRNA. The mRNA abundance of target genes and the housekeeping gene* GAPDH* was quantified by real-time PCR with an ABI 7300 (Applied Biosystems Foster City, CA, USA) using a Quanti Tect SYBR Green PCR Kit (TaKaRa Shuzo Co. Ltd., Kyoto, Japan). The quantitation of the target gene expression was evaluated by the 2-step method (Delta Delta Ct) [23]. The* GAPDH* gene was used as the internal standard to normalize the target gene level. Sequences for* CYP19A1* primers were GACCAATGAATCGGGCTATGT and TCTGTGGAAATCCTGCGTCTT. Sequences for* GAPDH* primers were GGAGTCCACTGGCGTCTTCA and ATGAGTCCTTCCACGATACCAA.

### 2.6. Western Blot Analysis

Thirty micrograms of each protein sample were separated by SDS-PAGE and transferred onto nitrocellulose membranes (Bio-Rad Laboratories, Inc., Hercules, CA, USA). The membranes were blocked for 1 h in Tris-buffered saline containing 0.01% Tween 20 with 5% nonfat dried milk. The membranes were then incubated overnight at 4°C with the relevant antibodies. The following antibodies were used in this study: monoclonal antiphospho-Smad3 (Ser423/425); monoclonal anti-Smad3 and polyclonal anti-Smad4 (Cell Signaling Technology Inc., Beverly, MA, USA); polyclonal anti-P450arom (BioVision Inc., California, USA); and polyclonal LaminA/C and* GAPDH* (SantaCruz Biotechnology Inc., SantaCruz, CA, USA). The membranes were washed and then incubated with the secondary peroxidase-conjugated anti-IgG (SantaCruz) antibody for 1 h. The immunoreactive proteins were detected using Enhanced Chemiluminescence reagents (Millipore, Billerica, MA, USA) followed by exposure to the detection system (Alpha Innotech, San Leandro, CA, USA). Quantity One Analysis software (Bio-Rad Laboratories Inc.) was used to determine protein density levels.

### 2.7. Measurement of Estradiol

ESCs (2 × 10^5^ viable cells) were cultured in 24-well plates. After treatment, the levels of E_2_ in the culture medium were measured by the electrochemiluminescence immunoassay (ECLIA; Beckman Coulter Co., California, USA) according to the operation manual of the Beckman company. The ECLIA kit is able to detect steroid contents (E_2_ ≥ 10 pg/mL). The intra- and interassay coefficients of variation were 15% and 20% for E_2_ assays.

### 2.8. Immunofluorescence

Cells from different treatment groups were washed with PBS three times and then fixed in 4% paraformaldehyde for 30 min at room temperature. The samples were subsequently incubated in permeabilization buffer (0.5% Triton X-100 in 20 mM Hepes, 3 mM MgCl_2_, 50 mM NaCl, 300 mM sucrose, and 0.02% NaN_3_) for 30 min at 37°C. The cells were then blocked in 5% BSA for 1 h at room temperature. The cells were washed, and the primary antibody to P-smad3 or smad3 was then added (1 : 100 dilution in PBS); the cells were then incubated for 1–3 hours at 37°C. The slide was washed three times with PBS, and then a fluorochrome conjugated second antibody (1 : 100 dilution in PBS) was added, after which the cells were incubated for 1 hour at 37°C. The cells were then washed with PBS and counterstained with 4′,6-diamidino-2-phenylindole (DAPI) (Vector Laboratories Inc., Burlingame, CA, USA) in the mounting medium. All steps using fluorochromes were performed under appropriate light conditions. The images were captured using a Leica fluorescence microscope (Leica, Heidelberg, Germany).

### 2.9. Targeted Knockdown of Smad 4

Two HP GenomeWide siRNA duplexes to Smad 4 (GenBank accession number NM_005359) were purchased from Invitrogen (Invitrogen): Smad4-1 (5′-UAUCCAUCAACAGUAACAAUAGGGC-3′) and Smad4-2 (5′-GCCCUAUUGUUACUGUUGAUGGAUA-3′). A control siRNA (Invitrogen) (5′-AATTCTCCGAACGTGTCACGT-3′) was used in all experiments. The Smad4 small RNA interference (RNAi) was named Smad4-siRNA, and the nonsense sequence to Smad4 was named negative control siRNA (NC-siRNA). The cells were transfected with siRNA duplexes using Lipofectamine™ 2000 Transfection Reagent (Invitrogen). All experiments were performed in duplicates using cells in 6-well culture plates at 70% confluence. Cells were cultured* in vitro* for 36 h and washed twice with PBS. Lipofectamine 2000 and siRNA were separately diluted in serum-free medium and incubated at room temperature for 5 minutes. They were then mixed and incubated at room temperature for additional 20 minutes. Aliquots of the transfection mixture were then added to cell culture dishes. The cells were cultured in the transfection media for 6 hours and then the culture medium was replaced with fresh Dulbecco modified Eagle medium F-12 (DMEM/F12; Sigma-Aldrich, St. Louis, Missouri, USA) medium containing 10% fetal bovine serum (FBS; Gibco, Australia). The cells were then cultured for 18 hours, after which they were treated with Activin A (25 ng/mL) for 24 h. The conditioned medium was removed and analyzed by Western blot for P450arom.

### 2.10. Statistical Analysis

All results in the study are shown as the mean ± standard deviation (SD). Each experiment was performed with triplicate samples in each treatment condition. Data distribution was evaluated by the Kolmorov-Smirnov normality test with Prism 4 computer software (GraphPad Software, La Jolla, CA, USA). After the normality test confirmed that the values did not depart significantly from the normal curve, they were analyzed by one-way analysis of variance (ANOVA) and by Fisher's least significant difference (LSD) test for multiple comparisons using SPSS 19.0 software (SPSS Inc., Chicago, Illinois, USA). *p* < 0.05 was considered statistically significant.

## 3. Results

### 3.1. Effects of Activin A on mRNA Level, Protein Expression, and Activity of P450arom in ESCs

**Figure 1:**
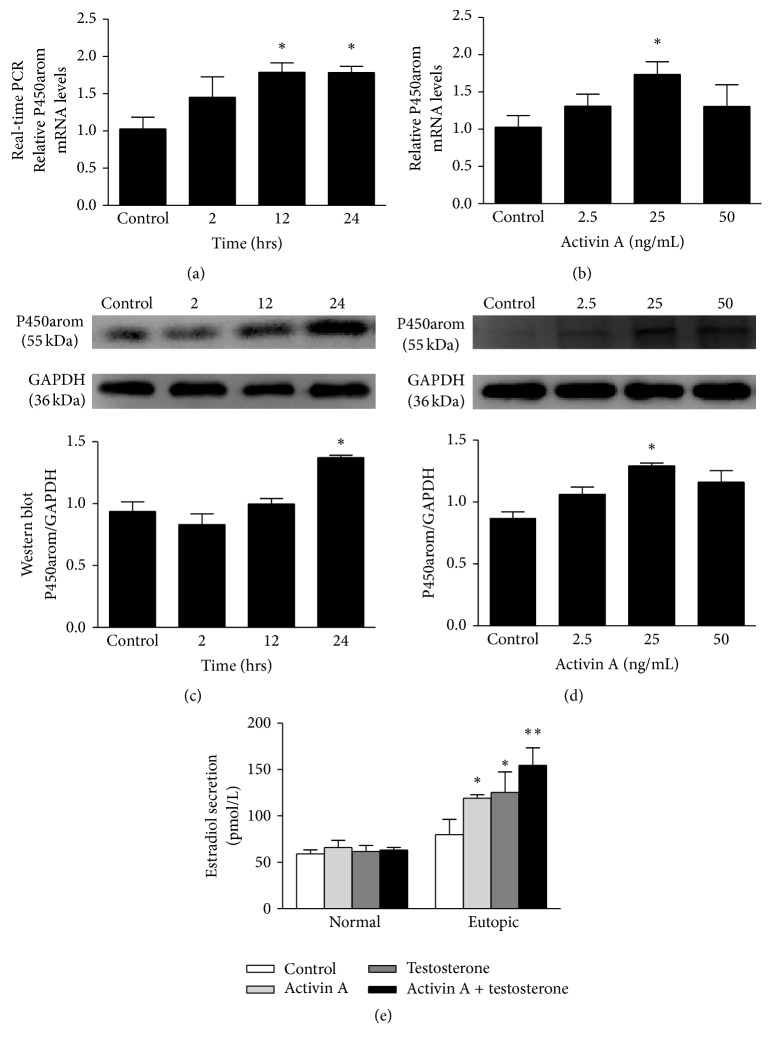
Effects of Activin A on mRNA level, protein expression, and activity of P450arom in ESCs. (a) Activin A time-course data (mean ± SD; *n* = 3) for P450arom mRNA expression. ESCs were treated with Activin A (25 ng/mL) for 2, 12, and 24 h and total RNA was isolated from untreated control and treated ESC. ^*∗*^ *p* < 0.05 versus control. (b) Activin A dose-response data (mean ± SD) of P450arom mRNA expression from three independent experiments performed in triplicate. ESCs were treated with Activin A (2.5, 25, and 50 ng/mL) for 24 h and total RNA was isolated from untreated control and treated ESC. ^*∗*^ *p* < 0.05 versus control. (c) Activin A time-course data (mean ± SD) for P450arom expression from three independent experiments performed in triplicate. The top panel of (c) shows representative data. The bottom panel of (c) is the mean ± SD data from three independent experiments performed in triplicate. ESCs were treated with Activin A (25 ng/mL) for 2, 12, or 24 hrs and total RNA was isolated from untreated control and treated ESC. ^*∗*^ *p* < 0.05 versus control. (d) Activin A dose-response data (mean ± SD) on P450arom expression from three independent experiments performed in triplicate. The top panel of (d) is representative data. The bottom panel of (d) is the mean ± SD from three independent experiments performed in triplicate. ESCs were treated with Activin A (2.5, 25, or 50 ng/mL) for 24 h and total RNA was isolated from untreated control and treated ESC. ^*∗*^ *p* < 0.05 versus control. (e) ESCs were incubated in the absence (control) or presence of Activin A (25 ng/mL) and/or testosterone (100 ng/mL). The estradiol concentrations in media secreted by stromal cells were measured in four groups. The data displayed in the figure show the mean ± SD from three independent experiments performed in triplicate. ^*∗*^ *p* < 0.05 versus control; ^*∗∗*^ *p* < 0.01 versus control.

Endometriosis is an estrogen-dependent disease. Therefore, we evaluated the effect of Activin A on the expression of P450arom, a critical enzyme for the local production of estrogens that drive the development of the disease [24]. As shown in Figure 1, we conducted time-course (2 h, 12 h, and 24 h) and dose-response (2.5 ng/mL, 25 ng/mL, and 50 ng/mL) experiments to determine the effect of Activin A on P450arom expression in ESCs from patients with endometriosis. As demonstrated in Figure 1, Activin A increased the P450arom mRNA (Figures 1(a) and 1(b)) and protein (Figures 1(c) and 1(d)) levels of ESCs in a time- and dose-dependent manner. The maximum increase of mRNA level was observed at the concentration of 25 ng/mL for 12 h. The maximum increase of protein level was observed at the concentration of 25 ng/mL for 24 h. These optimized conditions were used in subsequent experiments.

The concentrations of estradiol (E_2_) were measured to indirectly detect the P450arom activity. ESCs from normal endometria and patients with endometriosis were stimulated with Activin A (25 ng/mL) or testosterone (100 ng/mL) at 20°C for 24 h, and the concentrations of estradiol (E_2_) were measured. As depicted in Figure 1(e), the concentrations of estradiol in culture media from each treatment condition were detectable because the level was above the lower limits of the assay. Estradiol secretion in ESCs of subjects with normal endometria was not increased following Activin A and/or testosterone treatments. However, estradiol secretion in ESCs of patients with endometriosis was significantly increased following Activin A and/or testosterone treatments (^*∗*^ *p* < 0.05 versus control; ^*∗∗*^ *p* < 0.01 versus control). Collectively, our data show that Activin A increased the expression of P450arom, which is required for estradiol formation in ESCs. This elevation of estradiol following the P450arom increase suggests Activin mediated signal transduction may be involved in P450arom overexpression.

### 3.2. Effects of Activin A on the Smad Signaling Pathway in Eutopic Endometrial Stromal Cells (ESCs) from Patients with Endometriosis

**Figure 2:**
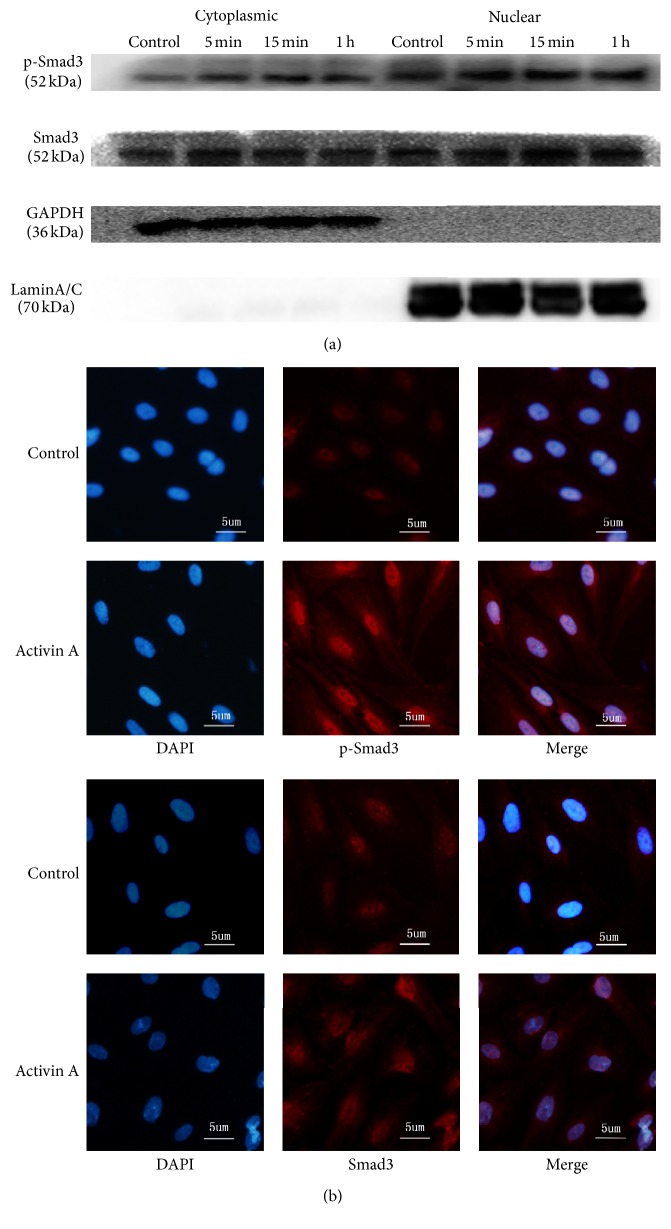
Effects of Activin A on the Smad signaling pathway in eutopic endometrial stromal cells (ESCs) from patients with endometriosis. (a) ESCs were treated with 25 ng/mL Activin A for the indicated times. The cell lysates were probed with antibodies against p-Smad3, Smad3, or GAPDH. A representative Western blot of p-smad3 and smad3 in cytoplasmic and nuclear fractions. (b) Immunofluorescence localization of Smad3 protein in eutopic endometrial stromal cells (ESCs) from a representative patient with endometriosis. Similar results were observed in other patients with endometriosis. DAPI stains the nuclei. FITC staining for p-Smad or Smads. The right column shows merged FITC/DAPI images. Control: no Activin treatment. Activin A: the cells were treated with 25 ng/mL of Activin A for 15 minutes. The top 6 images display the p-Smad-3 expression in ESCs. The bottom 6 images display Smad-3 expression in ESCs. Bar = 5 *μ*m.

ESCs were treated with 25 ng/mL of Activin A and analyzed at multiple time points. The cell lysates were probed with antibodies against p-Smad3, Smad3, or GAPDH.  Figure 2(a) shows a representative Western blot of p-smad3 and smad3 in cytoplasmic and nuclear fractions from three independent experiments. ESCs were treated with Activin A (25 ng/mL) for 5, 15, and 60 min. The treatments resulted in a significant increase of p-Smad3 in both the cytoplasm and nucleus. The amount of p-Smad3 also increased after treatment with Activin A for 5 minutes, and p-Smad3 reached a maximum at 15 minutes. The p-Smad3 levels then began to decrease after 1 hour. As illustrated in Figure 2(a), Smad3 expression was significantly increased as early as 15 minutes but slightly decreased by 1 hour only in nucleus. We found that Smad3 was constitutively expressed in the cytoplasm of ESCs. However, there is no significant difference in Smad3 expression at different time points.

Endometrial stromal cells express TGF-*β* receptors [25, 26] in the Smad signaling pathway. Therefore, we determined whether Activin A mediated its action through the activation of this pathway. The phosphorylation of Smad3 and its subsequent nuclear translocation are critical steps in the TGF-*β* signaling [27]. We examined the effects of Activin A on p-Smad3 nuclear translocation and accumulation. Our data indicated that phosphorylated Smad3 content was maximal at 15 min. Therefore, this time point was used in the subsequent experiments. The immunofluorescence data in Figure 2(b) show the localization of Smad3 protein in ESCs from a representative patient with endometriosis. Similar results were also found in other patients with endometriosis and all of the data were consistent with Western blot results (Figure 2(a)). Both p-Smad3 and Smad3 were primarily localized in the cytoplasm of untreated ESCs and it was suggested that the nuclear translocation was increased following Activin A treatment for 15 min. These results suggested that the effect of Activin A was partially mediated through the activation of the Smad pathway in the endometrium.

### 3.3. Effects of Activin A on Estradiol Secretion and Aromatase Expression in ESCs from Endometriosis Patients

**Figure 3:**
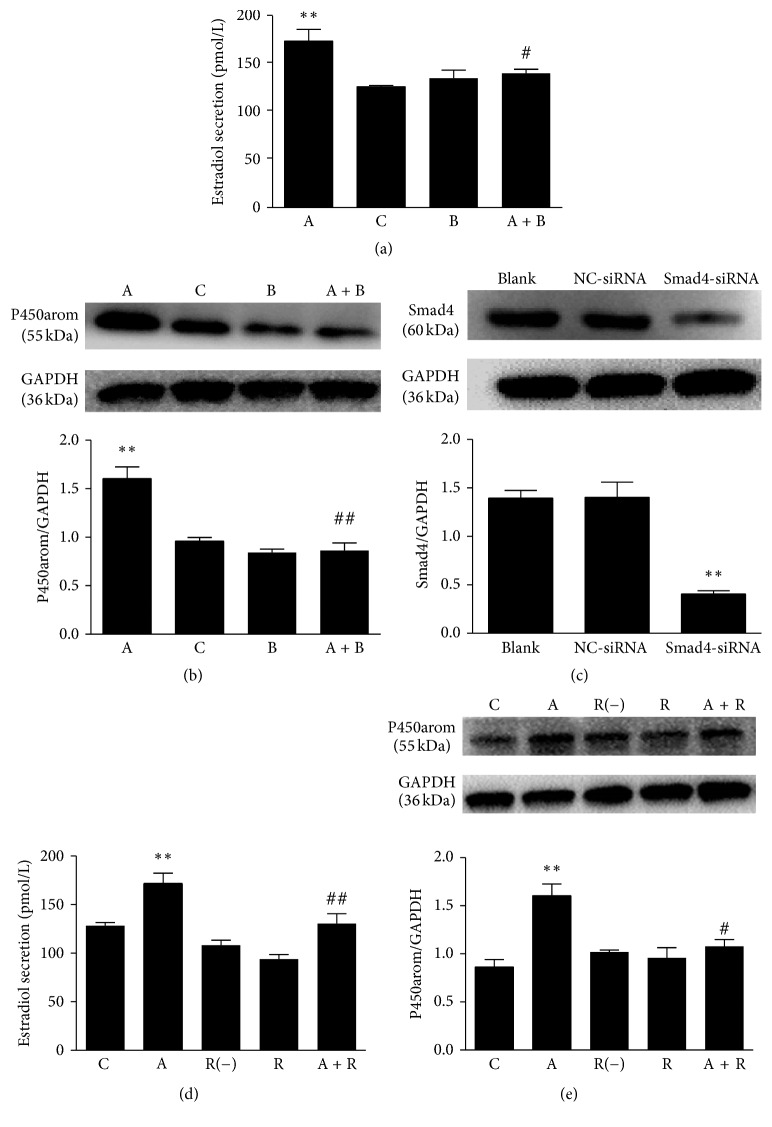
Effects of Activin A on estradiol secretion and aromatase expression in eutopic stromal cells (ESCs) from patients with endometriosis. C or blank: control; A: Activin A (25 ng/mL) for 24 hr; B: SB431542 (10 *μ*M) for 24 hr; A + B: 10 *μ*M SB431542 for 30 min followed by 25 ng/mL Activin A for 24 hrs; R: smad4-siRNA, R(−): NC-siRNA; A + R: transfected with Smad4-siRNA. The data are expressed as mean ± SD from three independent experiments performed in triplicate. ^*∗∗*^ *p* < 0.01 versus control; ^#^ *p* < 0.05 versus Activin A; ^##^ *p* < 0.01 versus Activin A. (a) The estradiol level was measured in various conditions. (b) P450arom expression was measured by Western blot (the top is representative and the bottom is the mean ± SD from three independent experiments performed in triplicate). (c) ESCs were transfected with Smad4-siRNA. The silencing effect of Smad4 protein expression was measured by Western blotting. (d) The estradiol level was measured in various conditions. (e) P450arom expression was measured by Western blot (top is a representative blot and the bottom is the mean ± SD from three independent experiments).

In the canonical Activin signaling pathway, the Activin ligands bind to a type II receptor serine/tyrosine kinase, which then phosphorylates a type I receptor (ALK4) [12]. The activated ALK4 then propagates intracellular signals, which are classically mediated by Smad proteins. SB431542 is a selective, potent small molecule inhibitor of ALK4, ALK5, and ALK7 [28]. As shown in Figure 3(a), Activin A increased estradiol secretion. In addition, the effect of Activin A was abolished by pretreating the cells for 30 minutes with SB431542. The effect of Activin A on the estradiol secretion suggested that signaling via one or more of these receptors might contribute to high P450arom expression and P450arom enzymatic activity.

To investigate whether Activin A affects the expression of P450arom through Smad proteins, we pretreated ESCs with SB431542 to block ALK4. As shown in Figure 3(b), the treatment with SB431542 significantly inhibited the expression of P450arom compared to Activin A treatment.

Smad4 is the only comediating Smad, and it is translocated to the nucleus with phosphorylated R-Smads. In the nucleus, the Smad complex subsequently modulates the transcription of Activin A target genes in mammals. To block the endogenous Activin-Smad signaling, we knocked down the level of Smad4 in ESCs by siRNA. ESCs were transfected with Smad4-siRNA in the subsequent experiment. The expression of Smad4 protein was analyzed by Western blot after transfection. As described in Figure 3(c), the level of Smad4 protein was decreased by approximately 71% by 48 hours after transfection with Smad4-siRNA. The effect of Activin-Smad signaling on steroidogenesis was then examined. As shown in Figure 3(d), the transfection of Smad4-siRNA reduced the production of E_2_. We knocked down the endogenous Smad4 using siRNA in ESCs to investigate whether Activin A requires Smad signaling to regulate the expression of P450arom. Compared with Activin A treatment, the knockdown of Smad4 significantly decreased the expression of P450arom (Figure 3(e)).

## 4. Discussion

P450arom may be involved in a positive feedback loop that favors the expression of key steroidogenic genes in endometriosis [8]. However, the precise expression level of P450arom in endometrium varies across published studies [29–36]. Numerous reports have confirmed the expression of P450arom in the endometrium at the mRNA transcriptional level [31, 32, 34, 37], at the protein translational level [30, 33, 35], and in enzymatic activity [35, 36]. However, Delvoux et al. [38] showed a complete absence of P450arom activity in endometrial samples. Moreover, a recent study reported that P450arom protein was not present in any type of endometriosis and that the mRNA of P450arom was barely detectable. These results suggest that locally produced P450arom (within endometrial lesions) may be less important in endometriosis development [39]. Consistent with previous results [30, 40], our data demonstrated that the P450arom expression was upregulated in both the eutopic endometrium and ectopic implants of women with endometriosis compared to those with normal endometria (Supplementary File, in Supplementary Material available online at http://dx.doi.org/10.1155/2016/5791510).

Although the pathologic mechanism of endometriosis remains uncharacterized and is regulated by multiple signaling pathways, Activin A-mediated activation of the Smad signaling may play a critical role in endometriosis. As described in Figure 2, Activin A strikingly activated the Smad pathway and induced Smad3 activation and nuclear translocation in ESCs. The treatment with either an inhibitor of ALK4 kinase or knockdown of Smad4 strongly inhibited the Smad3-induced nuclear translocation and P450arom activity. Previous studies have shown that Smads are low-affinity DNA-binding proteins and that the complex of Smad2/3/4 modulates gene expression by binding to Smad-binding elements (SBEs). SBEs are located within promoters containing numerous transcriptional coactivators and corepressors [8]. The consensus binding sequences for Smad proteins are 5-GTCT-3 and its complement, 5-AGAC-3 [41]. Aberrant P450arom expression in endometriosis is primarily mediated by the activation of promoter II [24]. There are several sequences of SBEs in the promoter II of P450arom. Therefore, we proposed that Activin A binds coactivators to mediate the transcription of P450arom in ESCs. The molecular mechanism of Activin A regulation of the P450arom expression in the nuclear compartment will be studied in future investigations.

Activin A has several important roles in cell proliferation, inflammation, immunity, and fibrosis [42–45]. Activin A can enhance the proliferation of stromal cells from endometriomas. Moreover, a comprehensive review indicated that Activin A played a complex role in controlling macrophage responses and was predominantly proinflammatory when acting early in an inflammatory response. However, there were anti-inflammatory effects when acting on already “inflamed” cells [46]. Ferreira et al. have shown that TGF-*β* exerts its actions in fibrosis by using Activin A as an intermediary [43]. In addition, Activin A increases matrix metalloproteinase (MMP) activity [47, 48] and promotes the invasion of endometrial cells into peritoneum* in vitro* [43]. A recent study has found that Activin A can stimulate human endometrial stromal cells to release interleukin-8 (IL-8) and vascular endothelial growth factor (VEGF), which may have implications for the pathogenesis of endometriosis [49]. Therefore, these results support several possible sites of Activin action involved in the pathogenesis of endometriosis.

Collectively, Activin A promotes the pathogenesis of endometriosis through at least three distinct processes. First, Activin A facilitates the process of endometrial cell invasion into the peritoneum to form implants. Second, Activin A can increase the secretion of estradiol by regulating the expression of P450arom via the ALK4-Smads pathway in endometrial cells. The combination of estrogen and dioxin (a highly toxic environmental contaminant derived from sources of 2,4,5-trichlorophenol) is involved in the pathogenesis of endometriosis by promoting chemokine secretion and invasion of endometrial stromal cells [50]. Third, Activin A stimulates inflammation and increases tissue fibrosis. Thus, the inflammatory status influences P450arom and steroid receptor expression in endometriosis [30].

## 5. Conclusions

The present data suggest that the mechanism of estrogenic synthesis is regulated by the Activin-Smad pathway in endometrial stromal cells. The Activin-Smad pathway may play an important role in P450arom expression and in the pathogenesis of endometriosis. The present study may supplement the estrogen-dependent theory of endometriosis and provides the experimental basis to explore new biological targets for the treatment of endometriosis.

## Supplementary Material

The protein and mRNA expression levels of P450arom from stromal cells in subjects with normal endometria and subjects with ovarian endometriosis were assessed by PCR and Western blot, respectively (Supplementary Figure). Figures A and C show representative data from three independent experiments. As shown in Figure B, the P450arom mRNA level was significantly increased in ectopic endometrial cells compared to normal and eutopic endometrial cells (*p*< 0.01). As illustrated in Figure D, the expression of P450arom protein in eutopic and ectopic tissues from patients diagnosed with endometriosis were significantly higher than in those with normal endometrial (*p*< 0.01). The difference in P450arom protein level between eutopic and ectopic tissues was apparent, but it was not statistically significant (*p* > 0.05).
