# Supplementary material for: Activin A Stimulates Aromatase via the ALK4-Smad Pathway in Endometriosis
Source: Biomed Res Int. 2016 Oct 19;2016:5791510. doi: 10.1155/2016/5791510 (PMC5090068; doi:10.1155/2016/5791510)
Supplement: Supplementary file 1 — The protein and mRNA expression levels of P450arom from stromal cells in subjects with normal endometria and subjects with ovarian endometriosis were assessed by PCR and Western blot, respectively (Supplementary Figure). Figures A and C show representative data from three independent experiments. As shown in Figure B, the P450arom mRNA level was significantly increased in ectopic endometrial cells compared to normal and eutopic endometrial cells (p< 0.01). As illustrated in Figure D, the expression of P450arom protein in eutopic and ectopic tissues from patients diagnosed with endometriosis were significantly higher than in those with normal endometrial (p< 0.01). The difference in P450arom protein level between eutopic and ectopic tissues was apparent, but it was not statistically significant (p > 0.05). [file 5791510.f1.doc]

**The expression of P450arom in stromal cells from subjects with normal endometrium and subjects with endometriosis.**

(A): A representative PCR data of P450arom expression in stromal cells from normal, eutopic or ectopic endometria. (B): Data displayed in the figure represent the mean +/- SD of P450arom (CYP19A1) mRNA levels from three independent experiments performed in triplicate for each group. (C): A representative western blot of P450arom protein in stromal cells from normal, eutopic or ectopic endometria. (D): The data displayed in the figure show the mean +/- SD of P450arom levels from three independent experiments performed in triplicate for each group. ***p* <0.01 versus normal endometrium; ^##^*p* <0.01 versus eutopic endometrium of the same patient.
